# High prevalence of epilepsy in two rural onchocerciasis endemic villages in the Mahenge area, Tanzania, after 20 years of community directed treatment with ivermectin

**DOI:** 10.1186/s40249-018-0450-3

**Published:** 2018-06-20

**Authors:** Bruno P. Mmbando, Patrick Suykerbuyk, Mohamed Mnacho, Advocatus Kakorozya, William Matuja, Adam Hendy, Helena Greter, Williams H. Makunde, Robert Colebunders

**Affiliations:** 1 National Institute for Medical Research, Tanga Research Centre, Tanga, Tanzania; 2 Global Health Institute, University of Antwerp, Antwerp, Belgium; 3 Muhimbili National Hospital, Dar es Salaam, Tanzania; 4 Enhance Tanzania Foundation, Dar es Salaam, Tanzania; 5 Muhimbili University of Health and allied Sciences, Dar es Salaam, Tanzania; 6 University of Texas Medical Branch, Galveston, TX USA

**Keywords:** Epilepsy, Onchocerciasis, Mahenge, Tanzania

## Abstract

**Background:**

Epilepsy is a neurological disorder with a multitude of underlying causes, which may include infection with *Onchocerca volvulus*, the parasitic worm that causes human onchocerciasis. A survey carried out in 1989 revealed a high prevalence of epilepsy (1.02% overall, ranging from 0.51 to 3.71% in ten villages) in the Mahenge area of Ulanga district, an onchocerciasis endemic region in south eastern Tanzania. This study aimed to determine the prevalence and incidence of epilepsy following 20 years of onchocerciasis control through annual community directed treatment with ivermectin (CDTI).

**Methods:**

The study was conducted in January 2017 in two suburban and two rural villages in the Mahenge area. Door-to-door household visits were carried out by trained community health workers and data assistants to screen for persons suspected of having epilepsy, using a standardised questionnaire. Persons with suspected epilepsy were then interviewed and examined by a neurologist for case verification. Onchocerciasis associated epilepsy was defined as epilepsy without an obvious cause, with an onset of seizures between the ages of 3–18 years in previously healthy children. In each village, fifty males aged ≥20 years were tested for onchocerciasis antibodies using an OV16 rapid test and were examined for presence of onchocerciasis nodules. Children aged 6–10 years were also tested using OV16 tests.

**Results:**

5117 individuals (median age 18.5 years, 53.2% female) from 1168 households were screened. 244 (4.8%) were suspected of having epilepsy and invited for neurological assessment. Prevalence of epilepsy was 2.5%, with the rural villages having the highest rate (3.5% vs 1.5%), *P* < 0.001. Overall incidence of epilepsy was 111 cases (95% *CI:* 73–161) per 100 000 person-years, while that of onchocerciasis associated epilepsy was 131 (95% *CI:* 70–223). Prevalence of OV16 antibodies in adult males and among children 6–10 years old was higher in rural villages than in suburban villages (76.5% vs 50.6, and 42.6% vs 4.7% respectively), (*P* < 0.001), while overall prevalence of onchocerciasis nodules was 1.8%.

**Conclusions:**

This survey revealed a high prevalence and incidence of epilepsy in two rural onchocerciasis endemic villages in the Mahenge area. Despite 20 years of CDTI, a high prevalence of OV16 antibodies in children aged 6–10 years suggests on-going *O. volvulus* transmission. Reasons for the persistence of on-going parasite transmission in the Mahenge area need to be investigated.

**Electronic supplementary material:**

The online version of this article (10.1186/s40249-018-0450-3) contains supplementary material, which is available to authorized users.

## Multilingual abstracts

Please see Additional file 1 for translations of the abstract into the six official working languages of the United Nations.

## Background

Epilepsy is a chronic neurological disease affecting an estimated 50 million people worldwide [1]. Prevalence rates vary geographically, with the highest being concentrated in low and middle income countries [1, 2]. Epilepsy presents with a variety of seizure types of different degrees of intensity, including nodding syndrome (NS), a rare form of the epilepsy which has been reported from Tanzania, Uganda and South Sudan [3–5]. NS is named after its characteristic seizure which causes loss of muscle tone in the neck and forward nodding of the head. Affected children may suffer from associated clinical manifestations such as mental retardation, loss of cognitive abilities and reduced growth rate [4]. Onset of NS occurs mainly in children aged 3–18 years [4].

Several studies have shown an association between onchocerciasis and epilepsy, and NS has only been reported from onchocerciasis endemic areas [6–8]. Case-control studies have found significantly higher rates of onchocerciasis infection in individuals with NS than in controls [4, 5]. However, it is not clear how the *Onchocerca volvulus* parasite is able to cause epilepsy [9]. Duke et al. previously noted the presence of small numbers of *O. volvulus* microfilariae (mf) in the cerebrospinal fluid (CSF) (< 2 mf/ml) in five of eight untreated heavily infected (> 100 mf/mg skin) onchocerciasis patients [10]. It has also been shown that the numbers of *O. volvulus* mf in the CSF increased up to 31 mf/ml during diethylcarbamazine treatment in 10 out of 11 heavily infected patients presenting with an ocular form of onchocerciasis. However, studies performed since the introduction of mass distribution of ivermectin have not been able to demonstrate the presence of mf in the CSF of patients with NS or other types of onchocerciasis associated epilepsy [9, 11, 12]. Moreover, a recent study suggests that NS is a disease caused by an autoimmune reaction to *O. volvulus* antibodies [13]*.*

Recent observations in the Democratic Republic of Congo (DRC) [14] and northern Uganda suggest that onchocerciasis control through mass drug administration (MDA) of ivermectin may decrease the incidence of NS and other forms of onchocerciasis associated epilepsy (OAE) [15].

The first cases of NS were reported among communities in the Mahenge Mountains in the 1960s [3]. In 1989, Rwiza et al. carried out a district-wide population-based survey aimed at determining the prevalence and incidence of epilepsy [16]. They documented a prevalence of active epilepsy of 1.02%, ranging from 0.51 to 3.71% in different villages, and a regional annual incidence of 73.3 new cases per 100 000 people [16]. Ivermectin has been distributed in the Mahenge area to control onchocerciasis since 1994 but annual community directed distribution of treatment with ivermectin (CDTI) started in 1997 [9].

The present study therefore aimed to investigate the effects of 20 years of onchocerciasis control through CDTI on the prevalence and incidence of epilepsy in selected villages in the Mahenge area of south eastern Tanzania.

## Methods

### Study site and population

**Fig. 1:**
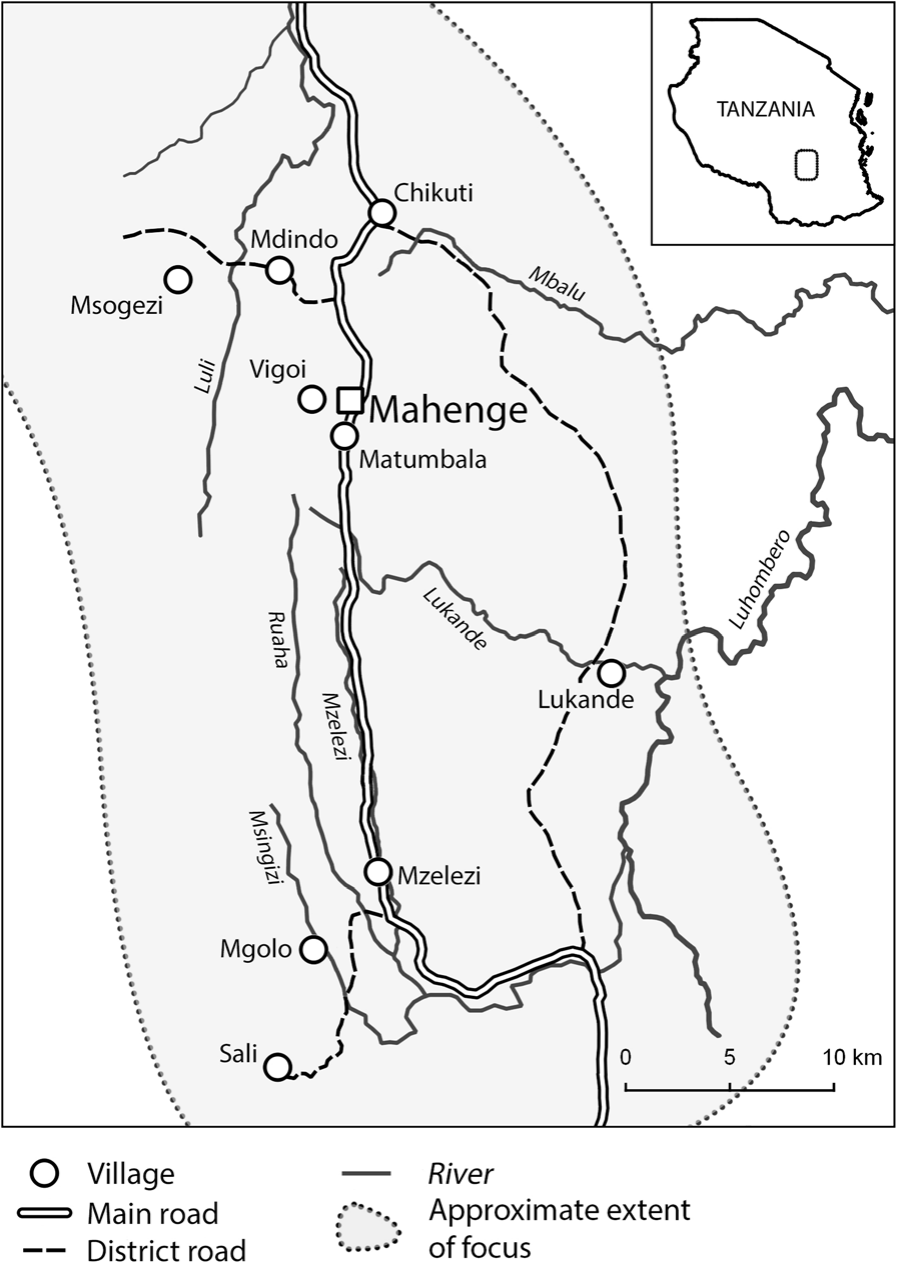
Map of the Mahenge area

Details of the study are described in a protocol paper published elsewhere [17]. In brief, investigations were carried out in the Mahenge area of Ulanga District, Morogoro region, south eastern Tanzania (Fig. 1). The area is mountainous with fast flowing rivers and streams which provide suitable breeding habitats for the blackfly vectors of onchocerciasis [18]. Occupations of the population include subsistence farming, livestock keeping (chicken, goats and pigs, the latter mainly kept in suburban villages) and working in gemstone mining. Study sites were selected based on the findings of Rwiza et al. in 1989 [16], and included villages with the highest epilepsy prevalence..These were two suburban villages (Matumbala and Vigoi) and two rural villages (Mdindo and Msogezi). Mdindo Village was the village with highest prevalence of epilepsy in the 1989 study by Rwiza et al. [16]. Matumbala was part of Vigoi village during the 1989 survey, but has since been separated, becoming an independent village in 2010. The suburban villages are located at an average altitude of 1050 m, while the rural villages are located at an average altitude of 590 m, and at a distance of 10 km and 17 km by road from Mahenge Township, respectively.

The Mahenge area is one of four onchocerciasis foci present in the Morogoro region. The others are in the Uluguru mountains, Kilosa, and the Nguru mountains [19]. The Mahenge focus was known for its high pre-control endemicity of onchocerciasis, which was first documented in the middle of last century [20]. MDA through annual CDTI began in the Mahenge area in 1997 [9], at which time the prevalence of disease through nodule examination was estimated to be 78.7% [21].

### Study design

This was a population based cross-sectional survey conducted in January 2017. The study involved all individuals within the households of the four study villages (Matumbala, Vigoi, Mdindo and Msogezi). During house-to-house visits, information was collected about geographical location (latitude and longitude) of the household, family size, age, gender, education level of children aged 7–10 years, signs of epilepsy among household members and history of death of individuals suspected to have epilepsy. All households and household members were provided with unique numbers which were used to identify them during epilepsy screening and onchocerciasis antibody testing.

### Epilepsy screening

Epilepsy case-finding was carried out using a two-stage approach. In the first stage, door-to-door household visits were performed by trained community health workers and data assistants, who asked a series of five questions to identify persons suspected of having epilepsy [22]. All inhabitants from the four villages were eligible for participation. It was asked whether a person: 1) had ever lost consciousness and experienced either loss of bladder control (1a) or foaming in the mouth (1b), 2) had ever experienced absence or sudden loss of contact with the surroundings for a short duration of time, 3) had ever experienced sudden uncontrollable twitching or shaking of the arms, legs or head, for a period of a few minutes, 4) had sometimes experienced sudden and brief bodily sensations, seen or heard things that were not there, or smelt strange odours, and 5) ever been diagnosed with epilepsy.

In the second stage, persons identified with suspected epilepsy were invited for an interview and clinical examination by a neurologist (MM) who was stationed in a central area in the village. Persons newly diagnosed with epilepsy and those with epilepsy who were not receiving treatment were referred to the epilepsy clinic at Mahenge hospital, or to the Msogezi dispensary which was also equipped to provide adequate epilepsy treatment and care.

### Definition of epilepsy and classification

Epilepsy cases were confirmed according to the International League Against Epilepsy criteria [23]. Nodding seizures were defined as episodes of reduced consciousness during which the head dropped forward repeatedly, and probable NS was defined according to the WHO case definition [24]. Onchocerciasis associated epilepsy was defined as a previously healthy person who had developed epilepsy without an obvious cause between the ages of 3 and 18 years [25].

### Assessment of the coverage of ivermectin at village level

Household members were asked about the intake of ivermectin during the most recent round of CDTI in 2016. In the absence of a household member, this information was obtained from the head of the household or the person responsible for the household at the time of visit. For children younger than 12 years, the information was verified by their parent or guardian.

### Determining onchocerciasis endemicity

The WHO rapid epidemiological monitoring of onchocerciasis (REMO) method was applied to determine the level of onchocerciasis endemicity in the study villages [26]. In each village, 50 adult males aged at least 20 years and resident in the community for at least 10 years, were invited to participate in the study. Each participant was examined for the presence of onchocerciasis nodules (subcutaneous nodules or deep, painless, firm, mobile nodules over bony prominences: pelvic girdle, costal grid, knees, skull), and a blood sample obtained by finger prick was tested for the presence of *O. volvulus* IgG4 antibodies using the OV16 antigen rapid test (Standard Diagnostics, Inc., Gyeonggi-do, Republic of Korea).

This test only identifies the presence of onchocerciasis antibodies, but is unable to discriminate between past exposure to the parasite and an active infection [27, 28]. All children aged 6–10 years were therefore invited to be tested for circulating *O. volvulus* antibody using the OV16 rapid test as an indirect indicator for recent transmission.

### Assessment of schooling

The level of education was assessed in all children aged between 7 and 10 years by asking which class the child was attending (the primary education in Tanzania is comprised of class one to seven). A child who was yet to be enrolled in primary school was graded as class zero. Information on whether a child had dropped out of the school was not determined.

### Data management and analysis

Data collection tools were developed in the open source software ‘Open Data Kit’ (ODK, https://opendatakit.org/) and data were collected using tablet computers and uploaded to a sever on a daily basis. All interviewers were trained to perform tablet-based surveys. They were familiar with mobile phones, short message service (SMS) text messaging, and the internet, and could easily follow the procedures of data collection and submission. A data coordinator was employed to check the completeness of the data entered in the tablets and to query any ambiguities that were immediately addressed the following day. Analysis was performed using STATA version 13 (STATA Corp Inc., TX, USA) and R version 3.3.2 (R Core Team [2017], Vienna, Austria). Epilepsy prevalence was calculated as the number of epilepsy cases per total number of people registered in the households visited. Incidence of new cases of epilepsy was defined as the number of persons who developed epileptic seizures within the 5 years preceding the survey, divided by the sum of population for the past 5 years assuming a growth rate (2.4%) in Morogoro region [29]. The deaths and migrations of new cases of epilepsy during this period were assumed to have a minimal effect on the incidence. The incidence of epilepsy was presented as cases per 100 000 person-years. A 5 year period was used due to the small number of new events occurring in a 1 year time period. Proportions were compared using a χ^*2*^-test, while means were compared using a *t*-test. Univariate logistic regression was used to assess the association between explanatory variables and epilepsy, whereas a multivariate model was used to control for confounding variables. The sensitivity of the five questions asked during the screening process was determined by the proportion of individuals identified as positive during screening, whose positive status was confirmed by the neurologist. Specificity was not determined since individuals who were identified as being negative were not invited for neurological examinations. Results are presented with accompanying 95% confidence intervals (95% *CI*), and *P*-values < 0.05 are deemed significant.

## Results

A total of 1168 households were visited and 5117 individuals (median age 18.5 years, 53.2% female) were registered, with Vigoi having the highest population (Table 1). Msogezi Village had the youngest population while Vigoi had the oldest population (Table 1). Most households (95%) depended on farming as their primary economic activity. Only a small proportion of households in the suburban villages (9.3% in Vigoi and 6.2% in Matumbala) were involved in other occupations including formal employment and mining. Out of 31 ethnic groups encountered in the study area, the predominant group was the Wapogoro (92.8%).

**Table 1:** Characteristics of the villages and population surveyed

Village	Mean altitude (range), m	No. of Households	No. of people	Median age (IQR)	No. aged ≥20 years (%)	No. aged 7–11 years (%)
Matumbala	1052 (786–1144)	244	972	20.1 (10.0–40.0)	337 (49.9)	71 (10.5)
Vigoi	1063 (504–1194)	388	1646	20.3 (10.5–40.4)	832 (50.6)	157 (9.5)
Mdindo	561 (444–802)	198	941	17.5 (8.5–38.1)	442 (47.0)	117 (12.3)
Msogezi	575 (491–731)	338	1558	15.4 (7.1–31.5)	661 (42.4)	221 (14.2)
Total		1168	5117	18.5 (8.7–37.5)	2272 (47.1)	566 (11.7)

*IQR*: Inter quartile range

### Epilepsy screening and confirmed cases

Table 2 shows the sensitivity of questions used for rapid screening of epilepsy. The screening questionnaire identified 244 (4.8%) individuals with suspected epilepsy. All were invited for neurological assessments and 239 (97.9%) were seen by the neurologist. Question 5 (Q5) “ever been diagnosed with epilepsy” had the highest sensitivity (86.4%), meaning it was the question most likely to result in a confirmed diagnosis, followed by Q1b), which was ‘whether a person had ever lost consciousness and experienced foaming in the mouth’. Question 4 was the least sensitive, which asked whether a person ‘had sometimes experienced sudden and brief bodily sensations, seen or heard things that were not there, or smelt strange odours’.

**Table 2:** Sensitivity of five screening questions together with their composite for epilepsy

Question	Number with positive response (%)	Number confirmed with epilepsy (%)	Sensitivity (95% *CI*)
Q1a. Lost consciousness and experienced loss of bladder control	75 (30.7)	51	68.0 (54.2–78.8)
Q1b. Lost consciousness and experienced foaming in the mouth	61 (25.0)	49	80.3 (70.1–90.6)
Q2. Experienced absence or sudden loss of contact with the surroundings for a short duration of time	152 (61.9)	103	67.8 (60.2–75.3)
Q3. Experienced sudden, uncontrollable twitching or shaking of arms, legs or head for few minutes	119 (48.8)	69	58.0 (49.0–67.0)
Q4. Experienced sudden and brief bodily sensations, seen or heard things that were not there, or smelt strange odours	76 (31.15)	36	47.4 (35.9–58.9)
Q5. Ever been diagnosed with epilepsy	110 (44.26)	95	86.4 (79.8–92.9)

Five individuals (a female aged 12 years from Mdindo, a female aged 4 and two males aged 28 and 55 years from Msogezi, and a female aged 25 years from Vigoi Village) did not appear for neurological assessment. None of the five individuals had been diagnosed previously to have epilepsy, three had a history of uncontrollable twitching or shaking of body parts, one had a history of both uncontrollable twitching or shaking of body parts and sudden loss of contact with the surroundings, while one girl aged 4 had a history of foaming in the mouth. The five individuals were not considered when calculating epilepsy prevalence.

Epilepsy prevalence was higher in the rural villages of Mdindo (3.5%) and Msogezi (3.5%) than in the suburban villages of Matumbala (1.6%) and Vigoi (1.4%), χ^*2*^ = 21.8, *P* < 0.001 (Table 3) but was similar in males and females.

**Table 3:** Individuals screened for epilepsy and prevalence of confirmed cases by gender, village and ethnic group

Village	Population enrolled						Epilepsy cases (%) by sex		
	Total	Males (%)	Suspected epilepsy (%)	No. examined (%)	Epilepsy cases (%)	χ^*2*^-test (*P*-value)	Male	Female	χ^*2*^-test (*P*-value)
Matumbala	972	431(44.3)	44 (4.5)	44 (100)	16 (1.65) 1.26^a^	–	7 (1.62)	9 (1.66)	0.002 (0.962)
Vigoi	1646	741(45.0)	50 (3.04)	49 (98.0)	23 (1.40) 1.26^a^	0.26 (0.612)	12 (1.62)	11 (1.22)	0.482 (0.487)
Mdindo	941	447(47.5)	56 (5.9)	55 (98.2)	33 (3.51) 3.71^a^	6.63 (0.010)	17 (3.80)	16 (3.24)	0.221 (0.638)
Msogezi	1558	774(49.7)	94 (6.0)	91 (96.8)	55 (3.53)	7.79 (0.005)	26 (3.36)	29 (3.70)	0.407 (0.523)
Ethnic group									
Wapogoro	4751		234 (4.9)		119 (2.51)				
Wangoni	54		1 (1.9)		1 (1.85)				
Wahehe	42		2 (4.8)		2 (4.76)				
Wandamba	32		1 (3.1)		1(3.13)				
Wandendeule	17		2 (11.8)		2 (11.77)				
Wazigua	9		2 (22.2)		2 (22.21)				
Others	212		4 (0.9)		0 (0)				
Total	5117	2393 (46.8)	244 (4.8)	239 (97.9)	127 (2.48)		61 (2.59)	66 (2.39)	0.132 (0.716)

^a^Epilepsy prevalence ratio observed in 1989 survey by Rwiza et al. where Matumbala was part of Vigoi Village

**Fig. 2:**
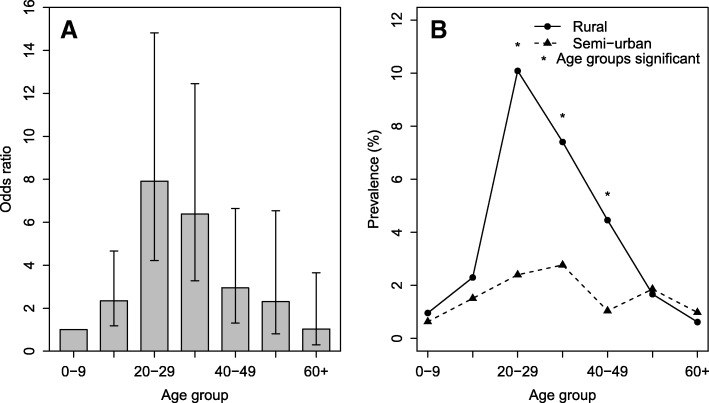
Risk of epilepsy adjusted by village (**a**) and prevalence of epilepsy by strata (**b**)

In a multivariate regression model, the risk of epilepsy increased with increasing age up to and including the age group 20–29 years, before declining thereafter (Table 4 and Fig. 2a). When analysis by age group was performed separately for rural and suburban villages, the prevalence of epilepsy was significantly different between the two settings in each age group between 20 and 29 and 40–49 years. Furthermore, individuals aged 20–29 years in the rural setting had the highest prevalence (10.1%), which declined progressively as age increased further i.e. beyond the 20–29 year old group (Fig. 2b).

**Table 4:** Univariate and multivariate regression models showing risks of epilepsy by age group village and strata

		Univariate		Multivariate	
Variable (N)	Epilepsy cases (%)	*OR* (95% *CI*)	*P-*value	*OR* (95% *CI*)	*P*-value
Age group					
0–9 (1466)	12 (0.82)	1		1	
10–19 (1229)	23 (1.87)	2.311 (1.145–4.663)	0.019	2.546 (1.259–5.146)	0.009
20–29 (722)	44 (6.09)	7.863 (4.127–14.984)	<0.0001	8.581 (4.494–16.387)	< 0.001
30–39 (559)	28 (5.01)	6.389 (3.226–12.656)	<0.0001	6.924 (3.487–13.750)	< 0.001
40–49 (491)	11 (2.44)	3.035 (1.355–6.802)	0.007	3.5 (1.555–7.862)	0.002
50–59 (281)	5 (1.78)	2.195 (0.767–6.28)	0.143	2.502 (0.872–7.179)	0.09
60+ (366)	3 (0.82)	1.001 (0.281–3.567)	0.998	1.118 (0.313–3.994)	0.964
Missing (5)	2 (40.0)				
Village					
Matumbala (972)	16 (1.65)	1		1	
Vigoi (1646)	23 (1.40)	0.847 (0.445–1.611)	0.612	0.837 (0.439−1.598)	0.589
Mdindo (941)	33 (3.51)	2.172 (1.187–3.972)	0.012	2.347 (1.277−4.314)	0.006
Msogezi (1558)	55 (3.53)	2.186 (1.246–3.838)	0.006	2.308 (1.309−4.071)	0.004
Strata					
Suburban (2499)	39 (1.49)	1			
Rural (2618)	88 (3.52)	2.414 (1.649–3.533)	< 0.001

Some households in the rural villages had more than one family member with epilepsy (five households in Mdindo had two family members; one household in Msogezi had three members, and two families had two members with epilepsy). All households with more than one person with epilepsy were of Wapogoro ethnicity with the exception of one household of Wazigua.

### Classification of epilepsy

The majority of the epilepsy cases (94 [74.0%]) were categorised as generalised tonic-clonic seizures Table 5. Probable NS was identified in 13 (10.2%) patients, of which 7 (53.9%) had a history of head nodding plus other forms of seizures; mainly generalised tonic-clonic seizures (*n* = 4).

**Table 5:** Frequency and type of seizure diagnosed

Type of Seizure	Frequency	%
Generalised tonic-clonic or atonic	94	74.0
Absences	9	7.1
Focal to bilateral tonic-clonic	8	6.3
Focal impaired awareness	3	2.4
Focal aware	2	1.6
Others	11	8.7
Total	127	100.0

**Fig. 3:**
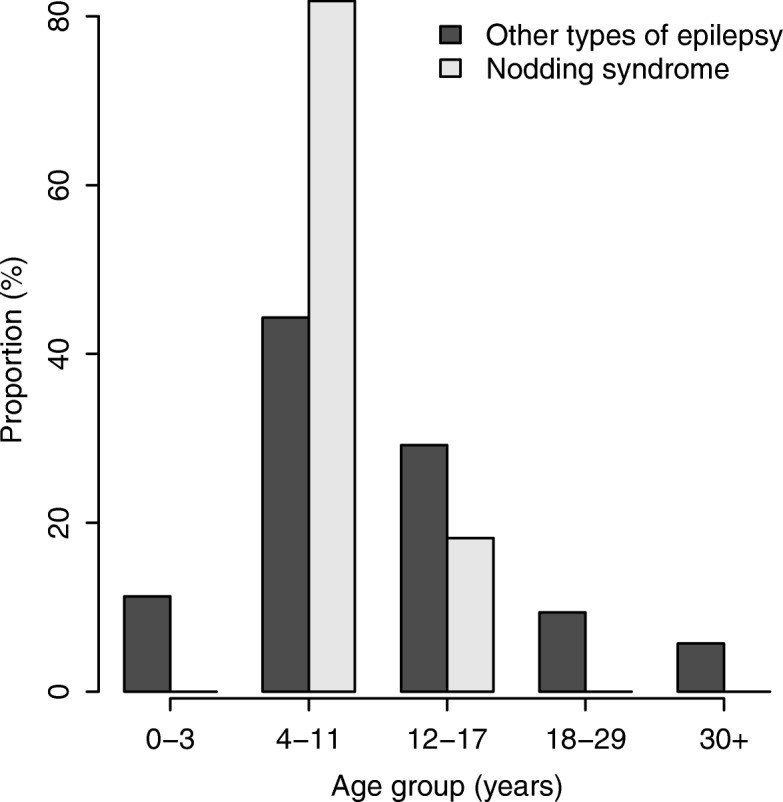
Distribution of age at onset of nodding syndrome and other types of epilepsy

Twelve (92%) of the persons with probable NS were from rural villages (nine [16.4%] out of 55 from Msogezi and 3 [9.1%] out of 33 from Mdindo). Only one (6.3%) out of 16 epilepsy patients from Matumbala was diagnosed with NS. The majority of epilepsy patients experienced their first seizure between the ages of 4–18 years, with a peak around 4–11 years (Fig. 3). The minimum age at onset of probable NS was 4 years, while that of other types of epilepsy was 2 years (Fig. 3).

### Incidence of epilepsy

Twenty seven participants experienced their first seizures in the 5 year period preceding the survey. The incidence of new epilepsy cases was therefore 111 (95% *CI:* 73–161) per 100 000 person-years for all forms of epilepsy (Table 6). Out of 27 new cases of epilepsy, 19 (70.4%) were individuals aged between 3 and 18 years. Six of these had a history of severe disease before the onset of the epilepsy, including: meningitis; malaria and coma; malaria and febrile seizures; malaria and meningitis; measles; and, psychomotor retardation. A total of 13 individuals in this age group (3–18 years) had no specific severe condition before the onset of the epilepsy and these were categorized as individuals with OAE, giving an incidence rate of 131 (95% *CI:* 70–223) cases per 100 000 person-years. The incidence of new cases of OAE was higher in the rural compared to suburban villages although this was not significant (rate ratio = 1.47, 95% *CI:* 0.48–4.48), (Table 6).

**Table 6:** Number of new cases of epilepsy, and incidence of epilepsy and of onchocerciasis associated epilepsy

	Overall Population				Population aged 3–18 years				
Village	Current population size (*n*)	Population total (2002–2007)	Total number of new cases	Incidence rate per 100 000 person-years (95% *CI*)	Population size (*n*)	Population total (2002–2007)	Total number of new cases	Total number of OAE cases	Incidence rate per 100 000 person-years (95% *CI*)
Matumbala	972	4635	4	86.3	376	1793	3	2	111.5
Vigoi	1646	7849	8	101.9	620	2956	5	3	101.5
Mdindo	941	4487	5	111.4	403	1922	4	3	156.1
Msogezi	1558	7429	10	134.6	686	3271	7	5	152.9
Total	5117	24 400	27	111 (73–161)	2085	9942	19	13	131 (70–223)

*OAE*: Onchocerciasis associated epilepsy; All estimates were calculated over a 5 year period

Msogezi village had the highest overall incidence of epilepsy while Matumbala had the lowest. Overall, the incidence was highest in individuals aged 10–19 years (187.7 per 100 000 person-years) followed by 0–9 years (171.7 per 100 000 person-years), 20–29 years (87.1 per 100 000 person-years) and 37.5 per 100 000 person-years in the age group 30–39 years. No new case was observed in individuals in the age group of 40 years or above.

### Use of ivermectin

Ivermectin intake during the past year was assessed in 4173 individuals aged above 6 years; 3329 (79.8%) of whom reported to have taken the drug during the last treatment round. The lowest rate was observed in Msogezi (76.0%), while the highest was recorded in Mdindo Village (87.3%). There was no difference in intake between persons with epilepsy (79.2%) and non-epileptic individuals (79.8%, χ^*2*^ = 0.03, *P* = 0.866), nor between males (80.8%) and females (78.9%, χ^*2*^ = 2.391, *P* = 0.122).

### Rapid epidemiological mapping of onchocerciasis

Two hundred and fifteen adult males (median age 40.2 years, IQR: 29.6–52.9) were examined for presence of onchocerciasis nodules (Table 7). Only five (2.3%) were found to have nodules; one from Matumbala, one from Vigoi, and three from Mdindo Village. One individual from Matumbala had two nodules, while the rest had one nodule only. The percentage of participants testing positive using the OV16 rapid test was significantly higher in the rural (76.5%) than in the suburban (50.6%) villages (*P* < 0.001).

**Table 7:** Prevalence of onchocerciasis nodules and OV16 antibodies among adult (≥ 20 years) males in suburban and rural villages

Village/Strata	Examined for nodules (REMO)		Nodules	OV16 tests		
	*N*	Median age (IQR)	No. positive (%)	Number screened (%)	Positive percentage (95% *CI*)	χ^*2*^-test (*P*-value)^δ^
Village						
Matumbala	35	38.5 (28.0–47.0)	1 (2.9)	34 (97.1)	32.3 (15.8–48.9)	
Vigoi	54	43.8 (33.5–55.5)	1 (1.9)	53 (98.1)	62.3 (48.8–75.8)	7.41 (0.006)
Mdindo	54	43.2 (30.8–53.5)	3 (5.6)	50 (92.6)	80.0 (68.5–91.5)	19.26 ( < 0.001)
Msogezi	72	35.7 (28.6–50.2)	0 (0)	69 (95.8)	73.9 (63.3–84.5)	16.42 (< 0.001)
Strata						
Suburban	126	42.5 (31.9–53.6)	2 (1.6)	119 (94.4)	50.6 (39.9–61.3)	
Rural	89	39.0 (28.9–51.9)	3 (3.4)	87 (97.6)	76.5 (68.7–84.2)	14.9 (< 0.001)
Total/Average	215	40.2 (29.6–52.9)	5 (2.3)	206 (95.8)	65.5 (59.0–72.1)	

^δ^Matumbala village was considered the baseline in all comparisons; REMO: Rapid epidemiological monitoring of onchocerciasis; *IQR* Inter quartile range

### Rapid assessment of risk of onchocerciasis transmission

**Fig. 4:**
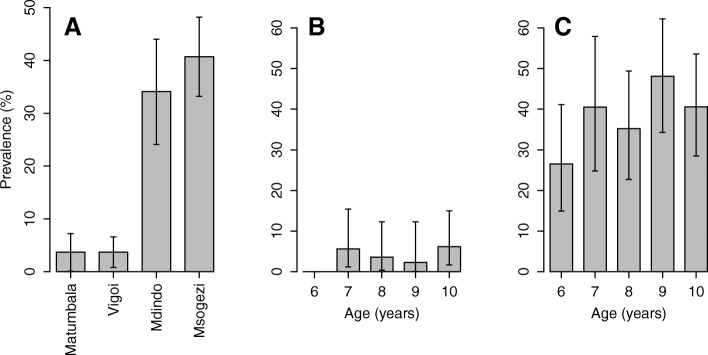
OV16 positivity rate in children 6–10 years by village (**a**), and age in urban (**b**) and rural (**c**)

Five hundred and thirty children aged 6–10 years were tested with the OV16 rapid test. The overall prevalence of OV16 positive children was 20.7%, and this was similar between females (20.1%) and males (21.3%, *P* = 0.73). The prevalence of positive OV16 tests was similar among the suburban villages (χ^2^ = 0.0004, *P* = 0.98), and also among the rural villages (χ^2^ = 1.102, *P* = 0.294) but was lower in the suburban (3.4%) than in the rural villages (38.4%), (χ^2^ = 97.6, *P* < 0.001) (Fig. 4a). None of the children aged 6 years from the suburban villages were positive for the OV16 tests, while in the older children the prevalence ranged from 2.3% (95% *CI:* 0.1–12.3) to 6.2% (95% *CI:* 1.7–15) (Fig. 4b). In the rural villages, children aged 6 years had the lowest prevalence of OV16 positive (26.5, 95% *CI:* 14.9–41.1) and those aged 9 years had the highest prevalence (48.1, 95% *CI:* 34.3–62.2) (Fig. 4c).

### Association between onchocerciasis and any form of epilepsy

**Fig. 5:**
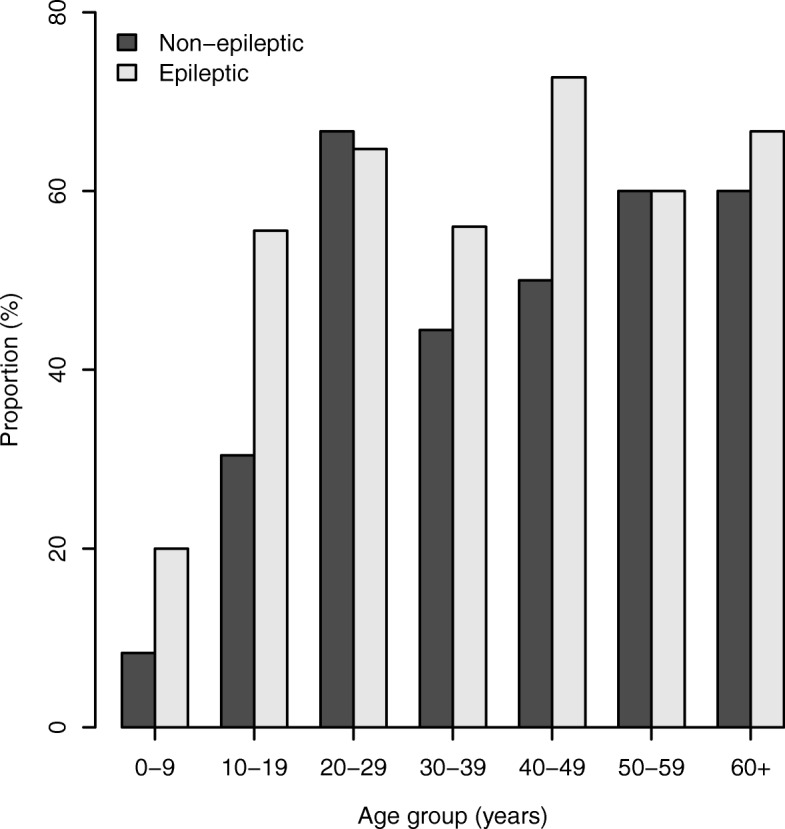
Distribution of OV16 positivity rate among individuals with confirmed and without epilepsy by age group

The prevalence of OV16 positivity rate was higher (57.9%) in individuals with confirmed epilepsy than in those without (41.25%), with an unadjusted odds ratio of 1.96 (95% *CI:* 1.09–3.53), *P* = 0.025 (Fig. 5). When age and village were included as covariates in the model, the level of significance for OV16 positivity among individuals with epilepsy was reduced slightly, with the odds ratio changing to 1.81 (95% *CI:* 0.93–3.49), *P* = 0.078. The association between OV16 and epilepsy was more evident in individuals aged below 20 years. In individuals aged 0–19 years, those with epilepsy were more likely to be OV16 positive, (*χ*^*2*^ = 2.87, *P* = 0.090).

### School attendance

**Fig. 6:**
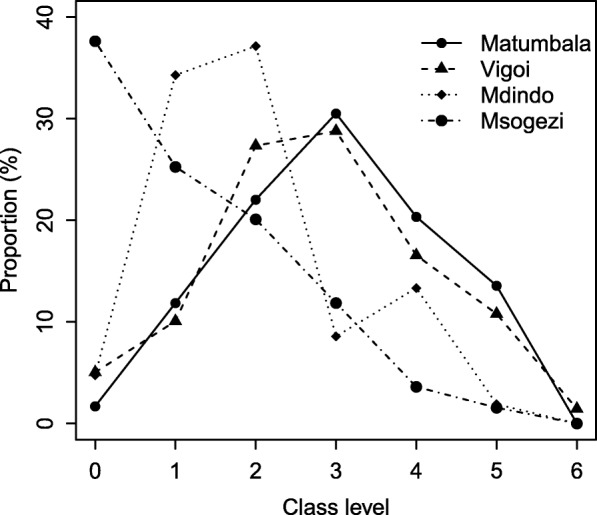
Proportion of children aged 7–10 years enrolled in primary school at different classes by village

Children from the rural villages often attended primary school at an older age than those in suburban villages, or were not attending school at all. 37.6% of the children from Msogezi were yet to start class one of primary school (Fig. 6). Five (83.3%) out of six children aged 7–10 years with confirmed epilepsy were yet to register for primary education, compared to 81 (15.6%) out of 519 children without epilepsy.

## Discussion

This study was aimed to determine the prevalence and incidence of epilepsy in the Mahenge area of Ulanga District, south eastern Tanzania, following 20 years of CDTI. The results show that despite the long term use of ivermectin there is still a high rate of *O. volvulus* transmission and high prevalence and incidence of epilepsy. This is especially true in the rural villages and confirms the observations of 30 years ago [16]. The prevalence of onchocerciasis nodules, however, was low.

The high prevalence of OV16 antibodies in children aged 6–10 years indicates on-going transmission of *O. volvulus* in these villages. This is consistent with entomological data collected in 2016 which showed that *O. volvulus* transmission by blackflies was continuing throughout the Mahenge mountains, with some lowland villages including Msogezi and Mdindo being among those most affected [30]. In the suburban villages, OV16 prevalence in children was less than 5%, which could be explained by low transmission rates since these villages are located at the top of the Mahenge mountains where fewer favourable blackfly breeding sites are located. The rural villages, which had an average OV16 prevalence in children of 38%, were located at medium altitudes where rapids and trailing vegetation provide suitable blackfly breeding sites among the numerous mountain rivers and streams. Of further interest was the low prevalence of nodules (only 2.3%) in men aged ≥20 years who had lived in the study area for over 10 years. This is likely to be due to the long term effects of CDTI in reducing community mf loads and alleviating clinical conditions associated with onchocerciasis, including the formation of nodules [31]. Substantial reductions in the risk of developing nodules have previously been observed after 5 or 6 years of annual CDTI [32–34]. It is also possible that smaller nodules could be located deeper in the subcutaneous tissues and were not palpated during physical examinations [35].

The majority of people with epilepsy and NS were identified in the rural villages (Mdindo and Msogezi) where the prevalence of OV16 antibodies was high among children and adults. This study underlines the association between onchocerciasis and epilepsy. The onset of seizures mainly occurred in children between the ages of 4 and 18 years. This age range is characteristic for persons with NS and onchocerciasis associated epilepsy [4]. The fact that minority ethnic groups were also affected by epilepsy and that epilepsy was clustered in the two rural villages, suggests that environmental exposure rather than a genetic factor was the main risk factor for the epilepsy.

The low level of schooling in the rural villages and among children with epilepsy may be related to lack of community motivation and commitment to education. Limited school supervision from higher levels due to poor accessibility as well as high incidence of epilepsy in the rural villages could be other contributing factors. In a study in northern Tanzania, behavioural problems, learning difficulties, parental stigmatization and teachers’ inadequate knowledge of seizure management were cited as the main barriers to education for young people with epilepsy [36]. The contribution of these factors among the children with epilepsy, in addition to poor access to quality education due to lack of classrooms and insufficient numbers of teachers, as well as the distance between households and schools which have been shown elsewhere [37] needs further investigation. Hunger could be another factor discouraging children from these poor communities to attend school. Most schools in these areas do not provide meals, despite the long distance the children have to walk each day [38].

Comparing the epilepsy prevalence and incidence rates with those observed by Rwiza et al. [16] before the implementation of CDTI is ambiguous. In their paper, Rwiza et al. only report the overall epilepsy prevalence and incidence rates in each age group, which makes it impossible to compare their results with our own. The overall, low prevalence (1.02%) and incidence rate (73.3 per 100 000 per person-years) in 1989 are due to the fact that more villages with lower prevalence were included in their study. In our study, we specifically included villages known to have a high prevalence of epilepsy based on the 1989 survey, in order to investigate the effect of CDTI on the incidence of epilepsy. Msogezi, a village with the highest prevalence in this survey was not part of the 1989 survey. Our study suggests, however, that there has been little change in the prevalence and incidence of epilepsy in the villages in the Mahenge area since 1989. The epilepsy prevalence rates in Mdindo (3.7% in 1989 vs 3.5% in 2017), and in Vigoi and Matumbala (1.3% in 1989 vs 1.5% in 2017) appear not to have changed over time. The fact that CDTI did not decrease the prevalence of epilepsy in the study villages is not too surprising. Indeed with increasing access to anti-epileptic treatment people with epilepsy remain alive and even with a low incidence of epilepsy the prevalence may remain high. However, after 20 years of CDTI it was expected to observe a shift in the distribution of epilepsy cases to older age groups, and to see a decrease in the number of incident cases of OAE. A shift to older age groups is difficult to assess, because Rwiza et al. did not report epilepsy prevalence per age group and village. The incidence of epilepsy (111 per 100 000 person-years) in the Mahenge villages in 2017 was approximately twice the incidence found in high income countries [39], but was comparable with the incidence of epilepsy reported by Rwiza et al. in 1989 (73.3 per 100 000 person-years) and with the incidence of epilepsy reported in studies in other African countries (64–187 per 100 000 person-years) [40]. The incidence of OAE in 2017 was 131 per 100 000 person-years. Unfortunately, incidence data of the study by Rwiza et al. are not available at village level which makes it impossible to compare the incidence between the two time points and to evaluate whether annual CDTI has had an effect on the incidence of epilepsy. However, in the rural villages a very high prevalence of epilepsy was observed in the 20–29-year-old age group (10.1%). This is in contrast with the suburban villages where there was no peak prevalence among 20–29 year olds. In the absence of CDTI, the peak prevalence of epilepsy in onchocerciasis endemic regions was observed in the 10–20-year-old age group [12, 41]. After several years of CDTI this peak prevalence switches to the 20–29 year age group [15]. The peak prevalence among 20–29 year olds in the rural Mahenge villages may be the consequence of a high incidence of OAE in these villages in the past, while the progressive decline in prevalence after the ages 20–29 suggests a very high mortality of epilepsy patients in the rural areas where less than 1% live to more than 60 years.

It is not clear why onchocerciasis transmission has not been interrupted in the Mahenge area despite of more than 20 years of CDTI. Approximately 78.9% of household members who were interviewed reported to have taken ivermectin in the year preceding the survey. However, this proportion is likely to be an overestimate because not each household member was interviewed. Household members not present during the survey may also have been absent during the last ivermectin distribution. Despite the fact that women are known to be more compliant in health interventions than men [42, 43], the rate of ivermectin intake was slightly lower in females than in males, although this was not significant. This could be due to the fact that the drug is not administered to pregnant and breast feeding mothers (during the first week of breast feeding). The clinical officer in-charge at the Msogezi dispensary indicated that the intake of ivermectin was very low in 2016 as the drug was distributed during the farming season when most people had already relocated to the farms. The WHO/MG/15.20 APO report in 2015 stated that in the Mahenge area (Morogoro CDTI area), the onchocerciasis elimination goal by 2025 will not be reached if no optimized CDTI or alternative treatments strategy is employed [44]. Many reasons may have contributed to the observed persistence of onchocerciasis transmission in the Mahenge focus, such as insufficient treatment coverage, programme supervision challenges, and poor compliance of communities due to low advocacy and low motivated drug distributors.

While many African countries have made significant progress to decrease onchocerciasis transmission in recent years, our results show the importance of strengthening onchocerciasis elimination programs, particularly in remote rural settings. Our study suggests that the CDTI programme in Mahenge has been functioning sub-optimally. This requires further investigation to establish the reasons as well as testing for alternative strategies for acceleration of onchocerciasis elimination. Alternative strategies may include biannual instead of annual distribution of ivermectin, which has been proposed by the WHO in situations where little progress is being made towards elimination. In Uganda for example, since the introduction of bi-annual CDTI and river larviciding in 2012, the interruption of onchocerciasis was successful in ten out of 17 foci [45], and no new cases of NS have been reported since 2013 [15]. Biannual ivermectin treatment appears to be a better option due to the rapid increase in skin mf, which occurs around 6 months after the administration of ivermectin [46]. Larviciding of rivers is another strategy that could supplement the current onchocerciasis control measures undertaken in Mahenge. The rural villages in the Mahenge area could be ideal study sites to evaluate, in a prospective way, the effects of an intensified onchocerciasis elimination programme on the incidence of epilepsy.

The high prevalence of epilepsy in the rural villages of the Mahenge signifies that treatment and care of epilepsy needs to be decentralized to the primary health care level. This will require organizing and training primary health care providers on how to diagnose and treat persons with epilepsy, and also training of teachers on how to take care of school children with epilepsy.

## Conclusions

Despite 20 years of CDTI, the prevalence and incidence of onchocerciasis associated epilepsy remains high in rural villages in the Mahenge area. The reasons for persistence of high prevalence of onchocerciasis in the Mahenge area need to be investigated.

## Additional file

Additional file 1: Multilingual abstracts in the six official working languages of the United Nations. (PDF 695 kb)

## Abbreviations

CDTI: Community directed treatment with ivermectin
*CI*: Confidence interval
CSF: Cerebrospinal fluid
DRC: Democratic Republic of Congo
IQR: Inter quartile range
MDA: Mass drug administration
NS: Nodding syndrome
OAE: Onchocerciasis associated epilepsy
ODK: Open Data Kit
*OR*: Odds ratio
REMO: Rapid epidemiological monitoring of onchocerciasis
WHO: World Health Organisation

## References

[1] World Health Organisation. Epilepsy 2017. http://www.who.int/mediacentre/factsheets/fs999/en/. Accessed 28 Sep 2017.

[2] Paul A, Adeloye D, George-Carey R, Kolčić I, Grant L, Chan KY (2012). An estimate of the prevalence of epilepsy in sub–Saharan Africa: a systematic analysis. J Glob Health.

[3] Aall-Jilek LM (1965). Epilepsy in the Wapogoro tribe in Tanganyika. Acta Psychiatr Scand.

[4] Dowell SF, Sejvar JJ, Riek L, Vandemaele KAH, Lamunu M, Kuesel AC (2013). Nodding syndrome. Emerg Infect Dis.

[5] Tumwine JK, Vandemaele K, Chungong S, Richer M, Anker M, Ayana Y (2012). Clinical and epidemiologic characteristics of nodding syndrome in Mundri County, southern Sudan. Afr Health Sci.

[6] Boussinesq M, Pion SDS, Kamgno J, Demanga-Ngangue A, Kamgno J, others (2002). Relationship between onchocerciasis and epilepsy: a matched case-control study in the Mbam Valley, Republic of Cameroon. Trans R Soc Trop Med Hyg.

[7] Kamuyu G, Bottomley C, Mageto J, Lowe B, Wilkins PP, Noh JC (2014). Exposure to multiple parasites is associated with the prevalence of active convulsive epilepsy in sub-Saharan Africa. PLoS Negl Trop Dis.

[8] Pion SDS, Kaiser C, Boutros-Toni F, Cournil A, Taylor MM, Meredith SEO (2009). Epilepsy in onchocerciasis endemic areas: systematic review and meta-analysis of population-based surveys. PLoS Negl Trop Dis.

[9] König R, Nassri A, Meindl M, Matuja W, Kidunda AR, Siegmund V (2010). The role of Onchocerca volvulus in the development of epilepsy in a rural area of Tanzania. Parasitology.

[10] Duke BO, Vincelette J, Moore PJ (1976). Microfilariae in the cerebrospinal fluid, and neurological complications, during treatment of onchocerciasis with diethylcarbamazine. Tropenmed Parasitol.

[11] Winkler AS, Friedrich K, Velicheti S, Dharsee J, König R, Nassri A (2013). MRI findings in people with epilepsy and nodding syndrome in an area endemic for onchocerciasis: an observational study. Afr Health Sci.

[12] Colebunders R, Tepage F, Rood E, Mandro M, Abatih EN, Musinya G (2016). Prevalence of river epilepsy in the Orientale Province in the Democratic Republic of the Congo. PLoS Negl Trop Dis.

[13] Johnson TP, Tyagi R, Lee PR, Lee MH, Johnson KR, Kowalak J (2017). Nodding syndrome may be an autoimmune reaction to the parasitic worm Onchocerca volvulus. Sci Transl Med.

[14] Levick B, Laudisoit A, Tepage F, Ensoy-Musoro C, Mandro M, Bonareri Osoro C (2017). High prevalence of epilepsy in onchocerciasis endemic regions in the Democratic Republic of the Congo. PLoS Negl Trop Dis.

[15] Colebunders R, Irani J, Post R (2016). Nodding syndrome, we can now prevent it. Int J Infect Dis.

[16] Rwiza HT, Kilonzo GP, Haule J, Matuja WBP, Mteza I, Mbena P (1992). Prevalence and incidence of epilepsy in Ulanga, a rural Tanzanian District: a community-based study. Epilepsia.

[17] Greter H, Mmbando BP, Makunde W, Mnacho M, Matuja W, Kakorozya A (2018). Evolution of epilepsy prevalence and incidence in a Tanzanian area endemic for onchocerciasis, and the potential impact of community-directed treatment with ivermectin: a cross sectional study and comparison over 28 years. BMJ Open.

[18] Hausermann W (1969). On the biology of Simulium damnosum Theobald, 1903, the main vector of onchocerciasis in the Mahenge Mountains, Ulanga, Tanzania. Acta Trop.

[19] Wegesa P (1970). Others. The present status of onchocerciasis in Tanzania. A review of the distribution and prevalence of the disease. Trop Geogr Med.

[20] Young WA, Farr AG, McKendrick AJ (1946). A report of the occurrence of onchocerciasis in Mahenge, Tanganyika, and in southern area of Lake Victoria. East Afr Med J.

[21] Tekle AH, Zouré HGM, Noma M, Boussinesq M, Coffeng LE, Stolk WA (2016). Progress towards onchocerciasis elimination in the participating countries of the African Programme for onchocerciasis control: epidemiological evaluation results. Infect Dis Poverty.

[22] Diagana M, Preux P-M, Tuillas M, Ould HA, Druet-Cabanac M (2006). Dépistage de l’epilepsie en zones tropicales: validation d’un questionnaire en Mauritanie. Bull Soc Pathol Exot.

[23] Fisher RS, Acevedo C, Arzimanoglou A, Bogacz A, Cross JH, Elger CE (2014). ILAE official report: a practical clinical definition of epilepsy. Epilepsia.

[24] World Health Organisation (2012). International Scientific Meeting on Nodding Syndrome. Meeting Report. UKaid, CDC, The Republic of Uganda, WHO, Kampala.

[25] Colebunders R, Njamnshi AK, van Oijen M, Mukendi D, Kashama JM, Mandro M (2017). Onchocerciasis-associated epilepsy: from recent epidemiological and clinical findings to policy implications. Epilepsia Open.

[26] Ngoumou P, Walsh JF, Mace JM (1994). A rapid mapping technique for the prevalence and distribution of onchocerciasis: a Cameroon case study. Ann Trop Med Parasitol.

[27] Weil GJ, Steel C, Liftis F, Li BW, Mearns G, Lobos E (2000). A rapid-format antibody card test for diagnosis of onchocerciasis. J Infect Dis.

[28] Lipner EM, Dembele N, Souleymane S, Alley WS, Prevots DR, Toe L (2006). Field applicability of a rapid-format anti-Ov-16 antibody test for the assessment of onchocerciasis control measures in regions of endemicity. J Infect Dis.

[29] NBS, OCGS. 2012 Population and housing census: population distribution by administrative areas. 2013. http://www.tzdpg.or.tz/fileadmin/documents/dpg_internal/dpg_working_groups_clusters/cluster_2/water/WSDP/Background_information/2012_Census_General_Report.pdf. Accessed 28 Sep 2017.

[30] Hendy A, Krüger A, Pfarr K, De Witte J, Kibweja A, Mwingira U (2018). The blackfly vectors and transmission of Onchocerca volvulus in Mahenge, south eastern Tanzania. Acta Trop.

[31] Kamga GR, Dissak-Delon FN, Nana-Djeunga HC, Biholong BD, Mbigha-Ghogomu S, Souopgui J (2016). Still mesoendemic onchocerciasis in two Cameroonian community-directed treatment with ivermectin projects despite more than 15 years of mass treatment. Parasit Vectors.

[32] Emukah EC, Osuoha E, Miri ES, Onyenama J, Amazigo U, Obijuru C (2004). A longitudinal study of impact of repeated mass ivermectin treatment on clinical manifestations of onchocerciasis in Imo state, Nigeria. Am J Trop Med Hyg.

[33] Katabarwa M, Eyamba A, Habomugisha P, Lakwo T, Ekobo S, Kamgno J (2008). After a decade of annual dose mass ivermectin treatment in Cameroon and Uganda, onchocerciasis transmission continues. Trop Med Int Heal.

[34] Ozoh GA, Murdoch ME, Bissek AC, Hagan M, Ogbuagu K, Shamad M (2011). The African Programme for onchocerciasis control: impact on onchocercal skin disease. Trop Med Int Heal..

[35] Albiez EJ (1983). Studies on nodules and adult Onchocerca volvulus during a nodulectomy trial in hyperendemic villages in Liberia and upper Volta. I Palpable and impalpable onchocercomata. Tropenmed Parasitol.

[36] Quereshi C, Standing HC, Swai A, Hunter E, Walker R, Owens S (2017). Barriers to access to education for young people with epilepsy in northern Tanzania: a qualitative interview and focus group study involving teachers, parents and young people with epilepsy. Epilepsy Behav.

[37] Hannum E (2003). Poverty and basic education in rural China: villages, households, and girls’ and boys’ enrollment. Comp Educ Rev.

[38] Omwami EM, Neumann C, Bwibo NO (2011). Effects of a school feeding intervention on school attendance rates among elementary schoolchildren in rural Kenya. Nutrition.

[39] Fiest KM, Sauro K, Wiebe S, Patten S, Kwon C, Dykeman J (2017). Prevalence and incidence of epilepsy: a systematic review and meta-analysis of international studies. Neurology.

[40] Ba-Diop A, Marin B, Druet-Cabanac M, Ngoungou EB, Newton CR, Preux PM (2014). Epidemiology, causes, and treatment of epilepsy in sub-Saharan Africa. Lancet Neurol.

[41] Kaiser C, Kipp W, Asaba G, Mugisa C, Kabagambe G, Rating D (1996). The prevalence of epilepsy follows the distribution of onchocerciasis in a west Ugandan focus. Bull World Health Organ.

[42] Hagström B, Mattsson B, Rost IM, Gunnarsson RK (2004). What happened to the prescriptions? A single, short, standardized telephone call may increase compliance. Fam Pract.

[43] Johansson E, Long NH, Diwan VK, Winkvist A (1999). Attitudes to compliance with tuberculosis treatment among women and men in Vietnam. Int J Tuberc Lung Dis.

[44] World Health Organisation (2015). Report of the consultative meetings on Strategic Options and Alternative Treatment Strategies for Accelerating Onchocerciasis Elimination in Africa African Programme for Onchocerciasis Control.

[45] The Carter Center. The Carter Cen. 2017. https://www.cartercenter.org/resources/pdfs/news/health_publications/river_blindness/rb-summary-2016.pdf. Accessed 1 Jan 2018.

[46] Cupp EW, Bernardo MJ, Kiszewski AE, Collins RC, Taylor HR, Aziz MA (1986). The effect of ivermectin on transmission of Onchocerca volvulus. Science (80- ).

