# Supplementary material for: High prevalence of epilepsy in two rural onchocerciasis endemic villages in the Mahenge area, Tanzania, after 20 years of community directed treatment with ivermectin
Source: Infect Dis Poverty. 2018 Jun 20;7:64. doi: 10.1186/s40249-018-0450-3 (PMC6009039; doi:10.1186/s40249-018-0450-3)
Supplement: Supplementary file 1 — Multilingual abstracts in the six official working languages of the United Nations. (PDF 695 kb) [file 40249_2018_450_MOESM1_ESM.pdf]

Translation of the abstract into the six official working languages of the United Nations

ارتفاع معدل انتشار الصرع في قريتين في المناطق الريفية مستوطنتين بداء العمي النهري في منطقة ماهينجي ، تنزانيا ، بعد 20 عامًا من العلاج المجتمعي الموجه بالإيفرمكتين.

قدمه: برونو بي ميمباندو، وباتريك سوكربوك، ومحمد مناشو ، وأدفوكاتوس كاكوروزيا ، ووليام ماتوجا، _و آدم هندي، وهيلينا جريتر، وويليامز هـ ماكوندي، وروبرت كليبندرز

الملخص

الخلفية: داء الصرع هو اضطراب عصبي يحتوي على العديد من الأسباب الكامنة ، والتي قد تشمل العدوى بكلابية الذنب الملتوية ، وهي الدودة الطفيلية التي تسبب داء العمى النهري البشري. وكشفت دراسة استقصائية أجريت في عام 1989 عن ارتفاع معدل انتشار الصرع (بنسبة 1.02 في المائة بشكل عام ، تتراوح بين 0.51 و 3.71 في المائة) في منطقة ماهينجي في مقاطعة أولانغا ، وهي منطقة مستوطنة لداء العمى النهري في جنوب شرق تنزانيا. تهدف هذه الدراسة إلى تحديد مدى انتشار ووقوع حالات الصرع بعد 20 عامًا من مكافحة داء العمى النهري من خلال العلاج المجتمعي الموجه بالإيفرمكتين (CDTI).

الطرق: أجريت الدراسة في يناير 2017 في ضاحيتين واثنتين من القرى الريفية في منطقة ماهينجي. تم القيام بزيارات منزلية من الباب إلى الباب من قِبَل عاملين صحيين مدرَّبين ومساعدي بيانات لفحص الأشخاص المشتبه في إصابتهم بالصرع باستخدام استبيان موحد. ثم تمت مقابلة وفحص الأشخاص الذين تم تشخيصهم بالاشتباه في الإصابة بالصرع من قِبَل طبيب أعصاب للتحقق من الحالة. تم تعريف داء العمى النهري المصاحب للصرع بأنه صرع دون سبب واضح مع ظهور نوبات بين سن 3-18 سنة في أطفال أصحاء سابقاً. تم اختبار خمسون من الذكور الذين تتراوح أعمارهم بين ≤y20 لكل قرية للأجسام المضادة لداء العمى النهري مع اختبار OV16 السريع وفحصت لوجود عقيدات داء العمى النهري. تم اختبار الأطفال الذين تتراوح أعمارهم بين 6-10 سنوات أيضاً باستخدام اختبارات OV16.

النتائج: تم فحص 5117 فرداً (متوسط العمر 18.5 سنة ، 53.2٪ من الإناث) من 1168 أسرة. تم الاشتباه في 244 (4.8٪) بإصابتهم بالصرع ودعوا لإجراء تقييم عصبي. كان معدل انتشار الصرع 2.5 ٪ ، ولدى القرى الريفية أعلى معدل (3.5 ٪ مقابل 1.5 ٪) ، القيمة الاحتمالية < 0.001. كان معدل حدوث الصرع الإجمالي 111 (95 ٪ فاصل ثقة : 73-161) لكل 100،000 شخص/ سنوات ، في حين أن داء العمى النهري المرتبط الصرع كان 131 (70-223). كان انتشار الأجسام المضادة OV16 في الذكور البالغين وبين الأطفال 6-10 سنوات أعلى في القرى الريفية من القرى في الضواحي (على التوالي 76.5 ٪ مقابل 50.6 ٪ ، و 42.6 ٪ مقابل 4.7 ٪) ، القيمة الاحتمالية < 0.001 ، في حين كان معدل انتشار عقيدات داء العمى النهري بشكل عام 1.8 ٪ .

الاستنتاجات: كشفت هذه الدراسة عن ارتفاع معدل انتشار وحالات الإصابة بالصرع في اثنين من القرى الريفية المستوطنة بداء العمى النهري في منطقة ماهينجي. على الرغم من مرور 20 عامًا على العلاج المجتمعي الموجه بالإيفرمكتين CDTI ، فإن الانتشار العالي للأجسام المضادة لـ OV16 في الأطفال 6-10 سنوات في هذه القرى يشير إلى انتقال داء العمى النهري المستمر. يجب التحقق من أسباب استمرار ارتفاع معدل انتشار داء العمى النهري في منطقة ماهينجي.

Translated from English version into Arabic by Noor Alquraishi, proofread by Bashaier Allam, through

TRANSLATORS
WITHOUT BORDERS

实施伊维菌素社区定向治疗 20 年坦桑尼亚马亨盖地区两个盘尾丝虫病流行村庄的癫痫发病率仍很高

Bruno P Mmbando, Patrick Suykerbuyk, Mohamed Mnacho, Advocatus Kakorozya, William Matuja, Adam Hendy, Helena Greter, Williams H Makunde, and Robert Colebunders

摘要

引言：癫痫是一种神经系统疾病，有许多潜在病因，其中可能包括感染盘尾丝虫（能引起人类盘尾丝虫病）。1989 年的一项调查显示，坦桑尼亚东南部盘尾丝虫病流行地区——乌兰加地区马亨盖的癫痫发病率很高，总发病率为 1.02%（0.51–3.71%）。本研究旨在确定经过 20 年伊维菌素社区定向治疗（CDTI）控制盘尾丝虫病，该地区癫痫的流行率和发病率。

方法：本研究于 2017 年 1 月在马亨盖地区的两个郊区和两个农村进行。经过培训的社区卫生工作者和数据助理进行门到门的家访，使用标准问卷筛查癫痫疑似病例。其后对癫痫疑似病例进行访问，并由神经科医生进行病例诊断。盘尾丝虫病相关性癫痫定义为先前健康的 3–18 岁儿童，无明显原因引发癫痫发作。每个村对 50 名年龄≥ 20 岁的男性进行盘尾丝虫病抗体 OV16 快速检测，并检查是否存在盘尾丝虫病皮下结节。6–10 岁儿童也接受 OV16 检测。

结果：共筛查了 1168 户 5117 人，中值年龄为 18.5 岁，女性占 53.2%。244 人（4.8%）被怀疑患有癫痫并被邀请进行神经评估。癫痫患病率为 2.5%，农村患病率最高（3.5% vs 1.5%），$P <$ 0.001。总的癫痫发病率为 111/10 万人口/年（95%*CI*：73–161），而盘尾丝虫病相关癫痫的发病率为 131/10 万人口/年（95% *CI*: 70–223）。成年男性和 6–10 岁儿童 OV16 抗体阳性率，在农村比郊区高（76.5% vs 50.6%，42.6% vs 4.7%），P <0.001，而盘尾丝虫病结节的总体患病率为 1.8%。

结论：调查结果显示，马亨盖地区两个盘尾丝虫病流行村的癫痫流行率和发病率都很高。尽管 CDTI 已实行了 20 年，但这些村庄 6–10 岁儿童的高 OV16 抗体阳性率表明盘尾丝虫病还在继续传播。因此，需要调查马亨盖地区盘尾丝虫病持续高发的原因。

Translated from English version into Chinese by Translated by Xue-Jiao Ma, edited by Pin Yang

CHINESE CENTER FOR DISEASE CONTROL AND PREVENTION
NATIONAL INSTITUTE OF PARASITIC DISEASES

Forte prévalence de l'épilepsie dans deux villages ruraux dans la région d'onchocercose endémique de Mahenge en Tanzanie, après 20 ans de traitement à l'ivermectine sous directives communautaires.

Bruno P Mmbando, Patrick Suykerbuyk, Mohamed Mnacho, Advocatus Kakorozya, William Matuja, Adam Hendy, Helena Greter, Williams H Makunde et Robert Colebunders

Résumé

Contexte: L'épilepsie est un trouble neurologique dont les causes sous-jacentes sont multiples et peuvent comprendre l'infestation par *Onchocerca volvulus*, le ver parasite qui cause l'onchocercose chez l'homme. Une enquête menée en 1989 a révélé une forte prévalence de l'épilepsie (prévalence globale de 1,02 %, extrêmes de 0,51 et 3,71 %) à Mahenge, dans le district d'Ulanga, une région d'onchocercose endémique dans le sud-est de la Tanzanie. Cette étude avait pour but de déterminer la prévalence et la fréquence de l'épilepsie après 20 ans de lutte contre l'onchocercose au moyen du traitement à l'ivermectine sous directives communautaires (TIDC).

Méthodes: L'étude a été menée en janvier 2017 dans deux villages périurbains et deux villages ruraux de la région de Mahenge. Des visites de porte à porte ont été effectuées par des agents de santé communautaires et des assistants chargés de recueillir les données, afin de dépister les cas suspects d'épilepsie à l'aide d'un questionnaire standardisé. Ces personnes ont ensuite été interrogées et examinées par un neurologue afin de confirmer la présence d'une épilepsie. L'épilepsie liée à l'onchocercose a été définie comme une épilepsie sans cause apparente dont les crises débutent chez des enfants âgés de 3 à 18 ans, en bonne santé jusque là. Cinquante hommes âgés de 20 ans et plus ont été testés dans chaque village avec le test rapide OV16 afin de rechercher les anticorps visant l'onchocercose et ont fait l'objet d'un examen clinique recherchant la présence de nodules onchocerquiens. Le test rapide OV16 a également été administré à des enfants âgés de 6 à 10 ans.
Résultats: 5117 individus (âge moyen 18,5 ans, 53,2 % de femmes) ont été dépistés dans 1168 foyers. Une épilepsie a été suspectée chez 244 (4,8 %) d'entre eux, qui ont été invités à faire un bilan neurologique. La prévalence de l'épilepsie était de 2,5 % dans les villages ruraux les plus touchés (3,5 % contre 1,5 %, $P < 0{,}001$). L'incidence globale de l'épilepsie était de 111 cas (IC à 95 % de 73 à 161) pour 100 000 personnes par an, et celle de l'épilepsie liée à l'onchocercose de 131 (70-223). La prévalence des anticorps anti-OV16 chez les hommes adultes et les enfants âgés de 6 à 10 ans était plus élevée dans les villages ruraux que dans les villages périurbains (76,5 % et 50,6 % respectivement chez les adultes, 42,6 % et 4,7 % chez les enfants, $P < 0{,}001$), tandis que la prévalence globale des nodules onchocerquiens était de 1,8 %.
Conclusions: Cette étude a révélé une prévalence et une incidence élevées de l'épilepsie dans deux villages ruraux d'onchocercose endémique dans la région de Mahenge. Malgré 20 ans de TIDC, la forte prévalence d'anticorps anti-OV16 chez les enfants âgés de 6 à 10 ans de ces villages suggère que la transmission de l'onchocercose n'a pas cessé. Les raisons de la persistance d'une prévalence élevée de l'onchocercose dans la région de Mahenge doivent être élucidées.

Translated from English version into French by Karine H, proofread by Suzanne Assenat, through

TRANSLATORS
WITHOUT BORDERS

Высокая распространённость эпилепсии в двух сёлах с эндемией онхоцеркоза в районе Махенге, Танзания, после 20 лет лечения местного населения ивермектином.

Бруно П Ммбандо (Bruno P Mmbando), Патрик Суйкербуйк (Patrick Suykerbuyk), Мохамед Мначо (Mohamed Mnacho), Адвокатус Какорозя (Advocatus Kakorozya), Вильям Матуя (William Matuja), Адам Хенди (Adam Hendy), Хелена Гретер (Helena Greter), Вильямс Х Макунде (Williams H Makunde) и Роберт Коулбандерс (Robert Colebunders)

Аннотация

Исходные данные: Эпилепсия — это неврологическое расстройство со множеством первопричин, в том числе инфицирование *Onchocerca volvulus*, паразитическим червем,

вызывающим онхоцеркоз у людей. Исследование, проведенное в 1989 году, показало высокую распространённость эпилепсии (в целом 1,02%, в диапазоне от 0,51 до 3,71%) в районе Махенге области Уланга, регионе эндемии онхоцеркоза в юго-восточной части Танзании. Это исследование предназначалось для определения распространенности и частоты случаев заболевания эпилепсией после 20-летнего контроля онхоцеркоза с помощью лечения местного населения ивермектином (ЛМНИ).
Методы: Исследование было проведено в январе 2017 года в двух пригородах и двух сёлах в районе Махенге. Подомовые обходы проводились обученными медицинскими работниками и помощниками по сбору данных для выявления лиц, подозреваемых в наличии эпилепсии, с использованием стандартизированной анкеты. Затем лица, у которых было выявлено подозрение на эпилепсию, опрашивались и обследовались неврологом для подтверждения диагноза. Связанная с онхоцеркозом эпилепсия определялась как эпилепсия без очевидной причины с наступлением судорог в возрасте от 3 до 18 лет у ранее здоровых детей. По пятьдесят мужчин в возрасте ≥20 лет из каждой деревни проверяли на наличие антител к онхоцеркозу при помощи экспресс-теста OV16 и обследовали на наличие узелков онхоцеркоза. Детей в возрасте 6–10 лет также проверяли при помощи тестов OV16.
Результаты: Было обследовано 5 117 человек (средний возраст 18,5 лет, 53,2% женщин) из 1 168 домашних хозяйств. У 244 (4,8%) лиц было заподозрено наличие эпилепсии, и их пригласили для прохождения неврологической оценки. Частота случаев эпилепсии составила 2,5%, причем самый высокий показатель наблюдался в сёлах (3,5% по сравнению с 1,5%), $P < 0{,}001$. Общая частота случаев эпилепсии составила 111 случаев (95% *ДИ*: 73–161) на 100 000 человеко-лет, в то время как частота связанной с онхоцеркозом эпилепсии составила 131 случай (70–223). Частота случаев выявления антител при помощи теста OV16 у взрослых мужчин и среди детей в возрасте 6-10 лет была выше в сёлах, чем в пригородах (соответственно 76,5% по сравнению с 50,6% и 42,6% по сравнению с 4,7%), $P < 0{,}001$, в то время как общая частота случаев развития узелков онхоцеркоза составляла 1,8%.
Выводы: Это исследование показало высокую распространенность и частоту случаев эпилепсии в двух сёлах с эндемией онхоцеркоза в районе Махенге. Несмотря на 20-летнее ЛМНИ, высокая распространенность антител, выявленных при помощи теста OV16 у детей в возрасте 6–10 лет в этих сёлах, свидетельствует о продолжающейся передаче онхоцеркоза. Необходимо изучить причины продолжающейся высокой распространённости онхоцеркоза в районе Махенге.

Translated from English version into Russian by Oksana Weiss, proofread by Liudmila Tomanek, through

TRANSLATORS
WITHOUT BORDERS

Alta prevalencia de epilepsia en dos poblaciones rurales con oncocercosis endémica en el área de Mahenge, en Tanzania, luego de 20 años de tratamiento directo de la población con ivermectina.

Bruno P. Mmbando, Patrick Suykerbuyk, Mohamed Mnacho, Advocatus Kakorozya, William Matuja, Adam Hendy, Helena Greter, Williams H. Makunde,y Robert Colebunders

Resumen

Contexto: La epilepsia es un desorden neurológico causado por numerosos factores, entre los que puede estar la infección por *Onchocerca volvulus*, el parásito que causa la oncocercosis humana. Una encuesta realizada en el año 1989 reveló una prevalencia elevada de epilepsia (1.02% total con una variación de entre 0.51 y 3.71%) en el área de Mahenge del distrito de Ulanga, una región con oncocercosis endémica en el sudeste de Tanzania El objetivo de este estudio consistía en determinar la prevalencia y la incidencia de la epilepsia luego de 20 años de control de la oncocercosis por medio del tratamiento directo de la comunidad con ivermectina (CDTI por sus siglas en inglés).

Métodos: La investigación se realizó en enero del año 2017 en dos aldeas suburbanas y dos aldeas rurales en la zona de Mahenge. Trabajadores de salud comunitarios capacitados y asistentes para la toma de datos realizaron visitas domiciliarias con un cuestionario estandarizado para detectar personas suspectados de padecer epilepsia. A continuación, las personas suspectadas de epilepsia fueron entrevistadas y examinadas por un neurólogo para confirmar el diagnóstico. Se definió la epilepsia asociada a la oncocercosis como epilepsia sin una causa evidente, con la aparición de convulsiones entre los 3 y los 18 años en niños que hasta entonces eran saludables. Se analizaron 50 varones mayores de 20 años de cada aldea con la prueba rápida Ov16 para buscar anticuerpos contra la oncocercosis, y se los examinó para detectar la presencia de nódulos de oncocercosis. También se emplearon las pruebas OV16 en niños de entre 6 y 10 años.

Resultados: Se estudiaron 5117 individuos (con edades promedio de 18.5 años, de los cuales el 53.2% eran del sexo femenino) provenientes de 1168 hogares. Se encontraron 244 casos (4,8%) presuntos de epilepsia, que fueron invitados a realizar una evaluación neurológica. La prevalencia de epilepsia fue del 2.5%, con la mayor tasa presente en las aldeas rurales (3.5% contra 1.5%), $P < 0.001$. La incidencia total de epilepsia fue 111 (95% *CI*: 73–161) por 100000 años-persona, mientras que la epilepsia asociada a la oncocercosis fue 131 (70–223). La prevalencia de los anticuerpos OV16 en hombres adultos y en niños de 6 a 10 años fue mayor en las poblaciones rurales que en las suburbanas (76.5% contra 50.6% y 42.6% contra 4.7% respectivamente), $P < 0.001$, 001 mientras que la prevalencia total de nódulos de oncocercosis fue del 1.8%.

Conclusiones: Esta investigación demostró una elevada prevalencia e incidencia de la epilepsia sobre dos poblaciones rurales de la zona de Mahenge con oncocercosis endémica. A pesar de 20 años de CDTI, la elevada prevalencia de anticuerpos OV16 en niños de entre 6 y 10 años de estas poblaciones sugiere que la transmisión de la oncocercosis persiste. Las razones de la persistencia de la alta prevalencia de la oncocercosis en la zona de Mahenge deben ser investigadas.

Translated from English version into Spanish by Lidia Norese, proofread by Gemira MARTINEZ, through

TRANSLATORS
WITHOUT BORDERS
